# Knowledge, Perceptions, and Expectations of Dental Anesthesia Among Patients Attending the College of Dentistry Clinic in Hail, Saudi Arabia: A Cross-Sectional Study

**DOI:** 10.7759/cureus.99941

**Published:** 2025-12-23

**Authors:** Hanady S Alrasheedi, Anifah N Alshammari, Hussein A Marouf

**Affiliations:** 1 Department of Dentistry, University of Hail, College of Dentistry, Hail, SAU

**Keywords:** anesthetists, dentistry, knowledge, perception, perioperative care

## Abstract

Introduction: Anesthesia is unique because it is not a direct means of treatment; rather, it allows other physicians to do things that may treat, diagnose, or cure an ailment, which would otherwise be painful or complicated. In the preoperative period, there are a lot of goals of preparation, but one of the most important is to eliminate anxiety. The current study aims to assess the patients’ knowledge regarding the anesthetist's role and their knowledge about dental anesthesia.

Methods: It is a sectional questionnaire prepared at the Faculty of Dentistry, Hail University, Hail, Saudi Arabia. The study population was female patients. The questionnaire consisted of a general part with questions on demography for all participants and three other sections containing multiple-choice questions. The data were analyzed using the IBM SPSS Statistics software, version 22.0 (IBM Corp., Armonk, NY, USA), and Microsoft Excel (Microsoft Corp., Redmond, WA, USA)

Results: A total of 100 patients participated in the survey; all of them were females; 86% of the participants were Saudis. Patients preferred general anesthesia because it allowed them not to see things during surgery (34%). The major reason for preferring regional anesthesia was its safety (56%), while the major reason for its refusal was concern about numbness (40%). The majority of patients reported that there are no benefits from visiting the anesthesia room before surgery (71%) and did not think that all pain types can be treated with anesthesia (83%). Finally, 64% of participants reported that physicians are not responsible for anesthesia, and 71% did not believe in preoperative anesthesia precautions.

Conclusion: Many of the patients in our research were unfamiliar with the role of anesthesia, its many forms and procedures, and the role of anesthesiologists both inside and outside of the operating room. This may be accomplished by interacting with patients, using print and electronic media, and becoming acquainted with the patients prior to surgery.

## Introduction

Anesthesia is unique because it is not a direct means of treatment; rather, it allows other physicians to treat, diagnose, or cure an ailment that would otherwise be painful or complicated. The preoperative evaluation embraces assessing the patient's physiological and psychological condition, gathering information regarding medical, surgical, and medication history, conducting laboratory examinations, and detecting anesthesia-related hazards. During the preoperative phase, numerous preparatory objectives exist, with the paramount goal being the alleviation of anxiety [1, 2]. Numerous patients experience dread and anxiety in the preoperative phase due to insufficient information regarding possible complications related to anesthesia and surgery. Numerous studies have shown inadequate public understanding of anesthesia and the role of anesthetists [3, 4].

A study conducted by Krishnamurthy et al. [5] demonstrated that most participants were aware of one or more symptoms associated with anaphylaxis. However, none of the participants were aware of all the symptoms associated with anaphylaxis. Another study by Mohajer et al. [6] showed that a total of 744 responded to the questionnaire; 58% of the participants were deemed to have sufficient understanding of anesthesia, while 42% had insufficient knowledge; 69% of respondents agreed that anesthesia is safe, while 31% disagreed. Their study also showed a correlation between the education level and the knowledge about anesthesia and anesthetists.

One more study was done by Sagün et al. [7] using a questionnaire consisting of 21 questions to be filled out by the patients who applied to the anesthesia clinic. They reported that out of the 250 patients studied, 37.6% had secondary education, and the highest percentage belonged to the housewives (33.6%). This study found that having an anesthetic experience and a high level of education have a statistically significant effect on anesthesia knowledge.

The current study aims to evaluate the awareness of local female patients regarding the role of dental anesthesia and anesthesiologists and the need for educating them.

## Materials and methods

It is a cross-sectional questionnaire that was prepared at the Faculty of Dentistry, Hail University, Hail, Saudi Arabia. The population was female patients who attended the Faculty of Dentistry, Hail University, from May 1, 2020, to August 31, 2020. 

Our inclusion criteria were female patients who were aged ≥18 years, attended the Faculty of Dentistry at Hail University, and had undergone any dental procedure that required anesthesia. As the department only receives female patients, the sample included only female patients. We excluded female patients who were aged < 18 years, males, and patients who had undergone any dental procedure that did not require anesthesia.

Sampling procedure

We used random sampling. The study was open. The questionnaire was interviewer-administered to help the patients fill out the questionnaire. The questionnaire was in English, and no translation was needed (Appendix A). The questionnaire included a basic component with demographic questions for all participants, as well as three other parts with multiple-choice questions addressing discrete facts and demanding a "Yes," "No," or "Don't know" response. Patients were asked about their physical activity and smoking status, as both can affect patients' response to anesthesia. Socioeconomic status was defined as low if patients had limited financial resources (i.e., difficulty affording food, housing, healthcare, or education); middle if they had moderate income and resources (i.e., enough money for daily operations, paying expenses, and modest growth) and were not considered poor; or high if they had greater access to financial resources (i.e., involved large transactions and major investments). Patients were categorized with a high educational level if they attended a university or college, and a low educational level if they obtained a degree before university or college.

The first segment included a series of questions designed to measure patients' attitudes and understanding of anesthesia, the role of anesthetists and surgeons in patient care, and patients' recollection of who the anesthetist or surgeon was if they had previously had anesthesia and surgery. The second segment consisted of multiple questions to assess patients' knowledge about anesthesia. In addition to anesthetists' experience in dealing with patients who had no prior experience with anesthesia or surgery. The third section consisted of a series of questions directed at all participants, assessing their knowledge of the proposed surgical procedure, technique, and choice of anesthesia (regional or general); potential complications of anesthesia; and desire to meet with the anesthetist and learn more about anesthesia.

The questionnaire used in this study was adapted from previously published and validated instruments that assessed patients’ knowledge and perceptions regarding anesthesia and the role of anesthesiologists, including those by Mohajer et al. [6], Sagün & Birbiçer [7], and Krishnamurthy et al. [5]. Content validity was ensured through expert review by faculty members in anesthesia and dentistry who confirmed the relevance and clarity of each item. Linguistic clarity and logical sequencing of questions were verified prior to data collection. Although internal-consistency reliability (e.g., Cronbach’s alpha (α)) was not recalculated in this study, the questionnaire’s prior validation in the cited works provides established reliability. A limited pilot application within the same institutional setting was conducted to confirm item comprehension and feasibility before the main survey. The response rate was 95%.

Knowledge scores were dichotomized as follows: Sufficient knowledge: participants answering ≥ 50% of the knowledge items correctly; Insufficient knowledge: participants answered fewer than 50% correctly.

Data analysis

Data and statistical analyses were performed using IBM SPSS Statistics, Version 22.0 (IBM Corp., Armonk, NY, USA) and Microsoft Excel (Microsoft Corp., Redmond, WA) for data entry and verification. Descriptive statistics (frequency and percentage) summarized categorical variables. Associations between socio-demographic factors and knowledge level were examined using the chi-square (χ²) test. The level of statistical significance was set at p < 0.05, and results with p < 0.001 were considered highly significant. Effect magnitude for significant associations was interpreted using Cramer’s V or Phi (φ), where applicable. All analyses adhered strictly to standard cross-sectional analytical procedures.

## Results

Socio-demographic characteristics

A total of 100 patients participated in the survey. All of them were women who attended the Faculty of Dentistry, Hail University. The socio-demographic data of the selected study sample are presented in Table 1. Participants were grouped into five age categories; 22% of the participants were aged between 18 and 24 years, 23% between 25 and 30 years, 21% between 31 and 35 years, and 20% between 36 and 40 years, while the last group included those who were more than 40 years old. The majority of the participants were Saudis (86%), and the rest (14%) were 14 non-Saudis. According to their responses about the level of education, 57% had a higher level of education.

**Table 1:** Socio-demographic data of the study sample

Categories		Frequency (percentage)
Age (in years)	18-24	22 (22%)
	25-30	23 (23%)
	31-35	21 (21%)
	36-40	20 (20%)
	40+	14 (14%)
Educational level	High	57 (57%)
	Low	43 (43%)
Occupation	Housewife	43 (43%)
	Medical professional	3 (3%)
	Non-medical	15 (15%)
	Not working	20 (20%)
	Students	19 (19%)
Nationality	Non-Saudi	14 (14%)
	Saudi	86 (86%)
Socio-economic condition	High	7 (7%)
	Middle	65 (65%)
	Poor	28 (28%)
Smoking status	Currently smoking	1 (1%)
	Ex-smoking	8 (8%)
	Non-smoking	91 (91%)
Physical activity	Active	64 (64%)
	Inactive	36 (36%)
Frequency of hospital visits	Never go to the hospital	6 (6%)
	Once every 5 years	35 (35%)
	Once every 6 months	18 (18%)
	Once every month	9 (9%)
	Once every one year	32 (32%)
Anesthesia knowledge	Insufficient	69 (69%)
	Sufficient	31 (31%)
Source of knowledge	Media	38 (38%)
	Physician	21 (21%)
	Public awareness events	29 (29%)
	Teaching and schooling	12 (12%)
Total		100 (100%)

Regarding participants’ occupation, the majority were housewives, representing about 43% of the whole sample. On the other hand, only 3% were medical professionals, 15% were non-medical, and 19% were students. Of the participants, 65% were of moderate socio-economic status, while only 7% were high level and 28% were poor.

Regarding physical activity and smoking, the studied sample showed that 64% were doing physical activity, and in addition, 91% were non-smokers.

Visiting hospitals varied among participants, as 35% of them visited hospitals every five years, 32% visited hospitals every year, and 6% never visited hospitals before. On the other hand, only 9% of the participants visited hospitals every month.

Participants’ knowledge about anesthesia was sufficient in 31% of them and insufficient in 69%. Most of their knowledge was from the media 38% while physicians had a limited role, about 21%.

Knowledge about anesthesia and its types

According to this scoring system, 31% of respondents demonstrated sufficient knowledge, and 69% insufficient knowledge; 34% and 33% of participants preferred general anesthesia because of fear of seeing things during surgery and less risk, respectively. While only 10% of participants preferred it because of their relatives’ advice, 49% of participants refused general anesthesia due to the fear of not regaining consciousness, while 31% of patients refused it because of fear of death (Table 2).

**Table 2:** Participants’ preferences regarding general and regional anesthesia

Categories		Frequency (percentage)
Reasons for preferring general anesthesia	Advice of relatives	10 (10%)
	Fear of seeing things during surgery	34 (34%)
	Less risk compared to regional anesthesia	33 (33%)
	Other choices not offered	23 (23%)
Reasons for refusal of general anesthesia	Expensive	9 (9%)
	Fear of death	31 (31%)
	Sore throat	11 (11%)
	Will not regain consciousness	49 (49%)
Reasons for preferring regional anesthesia	Advice of relatives	20 (20%)
	Better post op pain control	18 (18%)
	Less expensive	2 (2%)
	Safe	56 (56%)
	Want to awake during surgery	4 (4%)
Reasons for refusal of regional anesthesia	Concern about backache	12 (12%)
	Concern about headache	13 (13%)
	Concern about numbness	40 (40%)
	Don't want to awake during surgery	29 (29%)
	Relatively bad experience	6 (6%)
Total		100 (100%)

Regarding regional anesthesia, the major reason for preferring it was safety for 56% of participants, while the major reason for refusal was concern about numbness after the procedure for 40% of the participants (Table 2).

**Figure 1:**
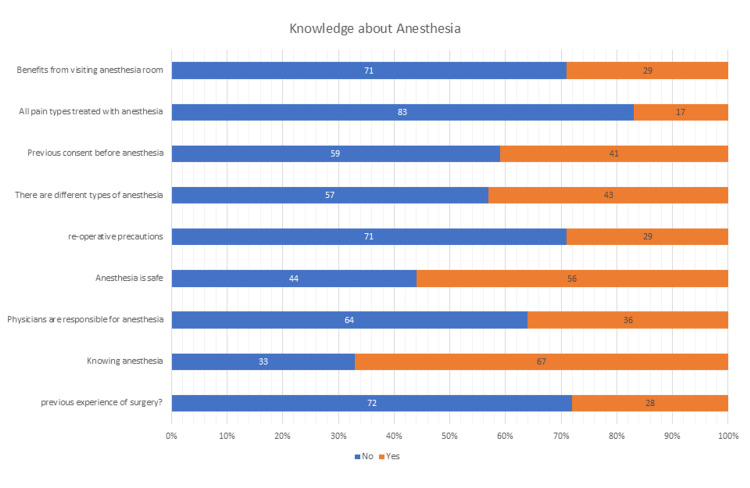
Participants' knowledge regarding anesthesia

Upon assessing knowledge regarding anesthesia, 71% of participants reported that there are no benefits from visiting the anesthesia room before surgery. Of all participants, 83% did not think that all pain types can be treated with anesthesia, and 59% did not know about the consent before anesthesia; 57% of participants did not think that there are different types of anesthesia, and 56% of them think that anesthesia is safe. 64% of participants said that physicians are not responsible for anesthesia, and 71% did not believe in preoperative anesthesia precautions, as shown in Figure 1.

**Figure 2:**
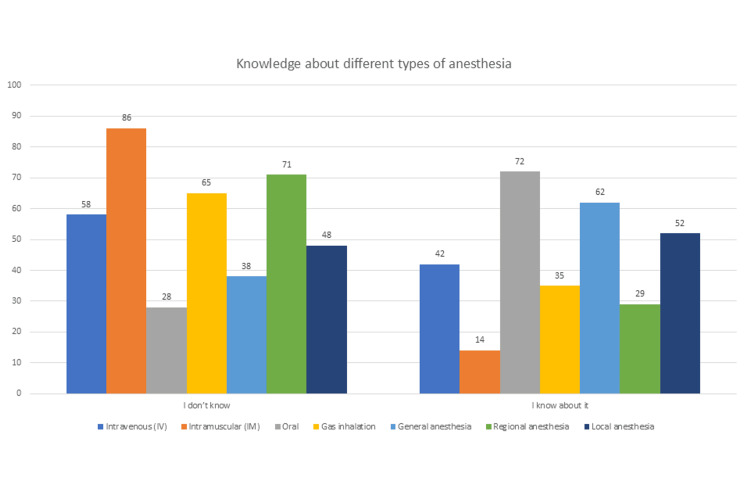
Participants' knowledge about different types of anesthesia

Finally, participants were asked about their knowledge of different types of anesthesia (Figure 2). Their answers have shown us that intramuscular anesthesia was the least common type among participants, as 86% of them did not know about it, followed by regional anesthesia. Participants were most familiar with oral anesthesia, as 72% of them knew well about it, followed by general anesthesia.

Correlation studies

Of participants with sufficient knowledge about anesthesia, 74.2% (n=23) had a high educational level, while only 25.8% (n=8) had a low educational level. On the other hand, 49.3% (n=34) of participants with insufficient knowledge had a high educational level, while 50.7% (n=35) of them had a low educational level; χ² value = 5.42; p-value = 0.02 < 0.05. It is a significant finding (Table 3).

**Table 3:** Comparison of sociodemographic characteristics according to anesthesia knowledge. *Statistical test applied = chi-square (χ²); **Not statistically significant (p > 0.05); ***Statistically significant (p < 0.05)' 0.0001*** highly significant (p < 0.001).

Categories		Anesthesia knowledge			
		Insufficient (n=69)	Sufficient (n=31)	P-value*	χ² value
Age (years)	18-25	15 (21.7%)	7 (22.6%)	0.795**	
	25-30	17 (24.6%)	6 (19.4%)		
	30-35	16 (23.2%)	5 (16.1%)		
	35-40	12 (17.4%)	8 (25.8%)		
	40+	9 (13.0%)	5 (16.1%)		
Educational level	High	34 (49.3%)	23 (74.2%)	0.02***	5.42
	Low	35 (50.7%)	8 (25.8%)		
Occupation	Housewife	34 (49.3%)	9 (29.0%)	0.029***	10.81
	Medical professional	0 (0.0%)	3 (9.7%)		
	Non-medical	8 (11.6%)	7 (22.6%)		
	Not working	13 (18.8%)	7 (22.6%)		
	Student	14 (20.3%)	5 (16.1%)		
Nationality	Non-Saudi	9 (13.0%)	5 (16.1%)	0.681**	
	Saudi	60 (87.0%)	26 (83.9%)		
Socio-economic condition	High	4 (5.8%)	3 (9.7%)	0.192**	
	Middle	42 (60.9%)	23 (74.2%)		
	Poor	23 (33.3%)	5 (16.1%)		
Previous experience of surgery?	No	54 (78.3%)	18 (58.1%)	.037***	
	Yes	15 (21.7%)	13 (41.9%)		
Physical activity	Active	39 (56.5%)	25 (80.6%)	0.02***	
	Inactive	30 (43.5%)	6 (19.4%)		
Frequency of hospital visits	Never go to the hospital	5 (7.2%)	1 (3.2%)	0.0001***	19.20
	Once every 5 years	33 (47.8%)	2 (6.5%)		
	Once every 6 months	6 (8.7%)	12 (38.7%)		
	Once every month	5 (7.2%)	4 (12.9%)		
	Once every one year	20 (29.0%)	12 (38.7%)		

Regarding occupation, of participants with sufficient knowledge about anesthesia, nine (29.0%) were housewives, three (9.7%) were medical professionals, seven (22.6%) did not work in the medical field, and five (16.1%) were students. Of participants with insufficient knowledge about anesthesia, 34 (49.3%) were housewives, 14 (20.3%) were students, eight (11.6%) did not work in the medical field, and none of them (0.0%) were medical professionals; χ² value = 10.81; p-value = 0.029 < 0.05 (Table 3).

Regarding hospital visit frequency, participants visiting hospitals every month, every six months, and every year comprised 28 (90.3%) of those with sufficient knowledge about anesthesia. Participants visiting hospitals every five years, every year, and no hospitals before, comprised 58 (84%) of those with insufficient knowledge about anesthesia; χ² value = 19.20; p-value = 0.0001 < 0.001 (Table 3).

## Discussion

When a dentist needs to inform patients of the benefits and risks of any procedure and the patient’s agreement to this procedure, this is where informed consent becomes evident. It has become mandatory for all dentists to obtain informed consent from every patient prior to any procedure because written and signed informed consent is the only evidence that can save the practitioners and their decision [8].

Results interpreted earlier had shown insufficient knowledge about anesthesia and anesthetists among patients of the Faculty of Dentistry, Hail University, in Hail City. Only 31% of our sample had sufficient knowledge about anesthesia, which is low compared to other studies. Another study in Saudi Arabia, Gazan [6], showed that 58.1% had sufficient knowledge about anesthesia, and about the same result was found in Pakistan [9] and in South Africa [10], which are higher than ours. Developed countries showed much higher results, such as 70% in Hong Kong [11]. Singh et al. conducted a study to evaluate the awareness among patients regarding anesthesia. The majority of their patients were not aware of the role of anesthesiologists inside and outside the operating room. However, they were aware of the general and regional anesthesia. Finally, they concluded that about half of the patients had good knowledge of anesthesia [12]. The reason for this low knowledge of patients regarding an anesthesiologist may be limited interaction of an anesthesiologist with their patients, as seen in hospital visits; participants visiting hospitals frequently knew more about anesthesia and anesthetists, and illiteracy.

Concerning anesthetists, only 36% in this study knew that anesthetists are physicians, but it is much higher in previous studies, such as 51% in Pakistan [9], 49.2% in Hong Kong [11], 40% in Turkey [12], and 87.4% in Saudi Arabia [6].

People may have concerns and fears about anesthesia. In this study, the major concern was not regaining consciousness in 49% of participants. A similar result found in a study in New York [13] was that 35% were afraid of not waking up after anesthesia.

The studied sample had a relatively low knowledge about anesthesia, as only 41% knew about the consent before anesthesia, but in a previous study in Saudi Arabia [6], about 78.5% knew about it. In addition, out of 100 participants in this study, only 29% knew about preoperative anesthesia measures. This result is low, comparable to 36.9% in India [14] and 91.9% in Saudi Arabia [6].

Regarding awareness about types of anesthesia, 43% of participants in this study were aware of different types of anesthesia, but in another previous study in India [15], only 7%. In addition, 62% of the study sample knew about general anesthesia. This is higher than some of the other studies, such as 27.5% in South Africa [10].

The level of education and occupation in our study was positively correlated with the knowledge about anesthesia and anesthetists (p-value = 0.02 < 0.05). This is similar to the results of other studies. Mohajer et al. [6] showed that the majority of the highly educated participants (82.2%) had sufficient knowledge about anesthesia. Marulasiddappa et al. [15] showed that patients with higher levels of education had better knowledge of anesthesiology and the role of an anesthesiologist (p < 0.05). In our study, previous surgery experience was positively correlated with knowledge of anesthesia (p-value = 0.037 < 0.05), but they were not correlated in Marulasiddappa et al.'s results [15], as they reported that about half of the participants had no idea about anesthesia despite the majority of them (62%) having had previous surgery with no statistically significant difference (p > 0.05) between those with previous surgery and those without previous surgery regarding their knowledge of anesthesiology and anesthesiologists.

Our study has its strengths. The possible outcomes patients might experience by having knowledge about anesthesia are alleviating fear of the unknown, reducing anxiety, increasing patients' satisfaction, and improving patients' compliance and adherence to the care plan. Our study has its own limitations. First, it was a single-center study that focuses mainly on the center and does not include other dental offices in the country. In addition, our study included only female participants, which can affect the generalizability of the study. Second, the small sample size and self-report bias can increase variability and lead to poor generalizability.

## Conclusions

Many of the patients in our research were unfamiliar with the role of anesthesia, its many forms and procedures, and the role of anesthesiologists both inside and outside of the operating room. Although this could be attributed to a lower level of education and less contact with physicians, the anesthesiologists' fraternity bears a significant responsibility to educate patients and surgeons about the role of anesthesia, its types, techniques, and benefits, and the critical role played by anesthesiologists both inside and outside of the operating room. This may be accomplished by interacting with patients, using print and electronic media, and becoming acquainted with patients before surgery. This might significantly improve the image of anesthesiologists among patients and the general public.

## Appendices

Appendix A

Questionnaire

Gender: Male /female

Age: 18-24/25-30/31-35/36-40/41 and above

Educational level: high/low

Occupation: medical professionals/ non-medical professionals/ students/ housewife/ not working

Nationality: Saudi/ Non-Saudi

Socio-economic condition: poor/ middle/ upper

Smoking: current smokers/ ex-smokers/ non-smokers

Physical activity: Active/ inactive

Anesthesia Knowledge: Sufficient/ insufficient

How frequently you go to the hospital: never go to the hospital/ once every month/ once every 6 months/ once every year/ once every 5 years

Source of knowledge: physician/ teaching and schooling/ media/ public awareness events

Reasons for preferring G/A: less risk/ fear of seeing things during surgery/ other choices not offered/ advice of relatives

Reasons for refusal of G/A: sore throat/ will not regain consciousness/ expensive/ fear of death

Reasons for preferring regional anesthesia: safe/ want to awake during surgery/ advice of relatives/less expensive/ want to see baby/better post op pain control

Reasons for refusal of regional anesthesia: concern about headache/concern about backache/concern about numbness/relative bad experience/don't want to awake during surgery

Previous experience of surgery: yes/ no

Knowing anesthesia: yes/ no

Physicians are responsible for anesthesia: 

Anesthesia is safe: yes/ no

Pre-operative precautions: yes/ no

There are different types of anesthesia: yes/ no

Previous consent before anesthesia: yes/ no

All pain types treated with anesthesia: yes/ no

Benefits from visiting the anesthesia room: yes/ no

Knowledge about the  route of administration:

Intravenous (IV): I know about it/ I don't

Intramuscular (IM): I know about it/ I don't

Oral: I know about it/ I don't

Gas inhalation: I know about it/ I don't

Knowledge about types of anesthesia: #

General anesthesia: I know about it/ I don't

Regional anesthesia: I know about it/ I don't

Local anesthesia: I know about it/ I don't
